# Interventions that effectively target *Anopheles funestus* mosquitoes could significantly improve control of persistent malaria transmission in south–eastern Tanzania

**DOI:** 10.1371/journal.pone.0177807

**Published:** 2017-05-18

**Authors:** Emmanuel W. Kaindoa, Nancy S. Matowo, Halfan S. Ngowo, Gustav Mkandawile, Arnold Mmbando, Marcelina Finda, Fredros O. Okumu

**Affiliations:** 1 Ifakara Health Institute, Environmental Health and Ecological Sciences Thematic Group, Morogoro, Tanzania; 2 University of the Witwatersrand, School of Public Health, Faculty of Health Science, Johannesburg, South Africa; 3 Wits Research Institute for Malaria, School of Pathology, Faculty of Health Sciences, University of the Witwatersrand, Johannesburg, South Africa; 4 Institute of Biodiversity, Animal Health and Comparative Medicine, G12 8QQ, University of Glasgow, Glasgow, United Kingdom; 5 Swiss Tropical and Public Health Institute, University of Basel, Basel, Switzerland; National Institute for Communicable Diseases, SOUTH AFRICA

## Abstract

Malaria is transmitted by many *Anopheles* species whose proportionate contributions vary across settings. We re-assessed the roles of *Anopheles arabiensis* and *Anopheles funestus*, and examined potential benefits of species-specific interventions in an area in south-eastern Tanzania, where malaria transmission persists, four years after mass distribution of long-lasting insecticide-treated nets (LLINs). Monthly mosquito sampling was done in randomly selected households in three villages using CDC light traps and back-pack aspirators, between January-2015 and January-2016, four years after the last mass distribution of LLINs in 2011. Multiplex polymerase chain reaction (PCR) was used to identify members of *An*. *funestus* and *Anopheles gambiae* complexes. Enzyme-linked immunosorbent assay (ELISA) was used to detect *Plasmodium* sporozoites in mosquito salivary glands, and to identify sources of mosquito blood meals. WHO susceptibility assays were done on wild caught female *An*. *funestus* s.l, and physiological ages approximated by examining mosquito ovaries for parity. A total of 20,135 *An*. *arabiensis* and 4,759 *An*. *funestus* were collected. The *An*. *funestus* group consisted of 76.6% *An*. *funestus* s.s, 2.9% *An*. *rivulorum*, 7.1% *An*. *leesoni*, and 13.4% unamplified samples. Of all mosquitoes positive for *Plasmodium*, 82.6% were *An*. *funestus* s.s, 14.0% were *An*. *arabiensis* and 3.4% were *An*. *rivulorum*. *An*. *funestus* and *An*. *arabiensis* contributed 86.21% and 13.79% respectively, of annual entomological inoculation rate (EIR). *An*. *arabiensis* fed on humans (73.4%), cattle (22.0%), dogs (3.1%) and chicken (1.5%), but *An*. *funestus* fed exclusively on humans. The *An*. *funestus* populations were 100% susceptible to organophosphates, pirimiphos methyl and malathion, but resistant to permethrin (10.5% mortality), deltamethrin (18.7%), lambda-cyhalothrin (18.7%) and DDT (26.2%), and had reduced susceptibility to bendiocarb (95%) and propoxur (90.1%). Parity rate was higher in *An*. *funestus* (65.8%) than *An*. *arabiensis* (44.1%). Though *An*. *arabiensis* is still the most abundant vector species here, the remaining malaria transmission is predominantly mediated by *An*. *funestus*, possibly due to high insecticide resistance and high survival probabilities. Interventions that effectively target *An*. *funestus* mosquitoes could therefore significantly improve control of persistent malaria transmission in south–eastern Tanzania.

## Background

Malaria continues to be one of the most significant mosquito-borne parasitic diseases, affecting about 212 million people, causing 429,000 deaths annually [1], and adversely affect socio-economic development in sub-Saharan African countries [2]. The World Health Organization (WHO) estimates that there has been a decline in malaria burden, and that morbidity worldwide reduced by 21% and mortality by 29% between 2010 and 2015, but sub-Saharan Africa still accounts for approximately 92% of all malaria deaths and cases [1]

Tanzania has experienced a decline in malaria transmission following the introduction of insecticide treated nets (ITNs) and the scale-up of long-lasting insecticide treated nets (LLINs) [3]. By 2010, the country had made significant progress, and most areas that were experiencing prevalence above 50% in 2000, now had below 10% prevalence [4]. However, the most recent malaria indicator survey has returned an average prevalence of 14.8% prevalence in children under 5 years in Tanzania [5]. Getting to zero transmission therefore remains a major challenge, mainly because the persistent malaria transmission is mediated by mosquitoes that are not adequately responsive to control by existing indoor insecticidal interventions, such as LLINs and indoor residual spraying (IRS). The Tanzanian National Malaria Control Program is aiming at cutting down malaria transmission to 1% by 2020 [6], thus the need for new complementary approaches is even more urgent.

Despite the high coverage of LLINs, high indoor densities of potentially infectious mosquitoes remain common in some places [7, 8]. Evidence suggests that indoor densities of major malaria vectors such as *An*. *funestus* and *An*. *arabiensis* depend on house design features, environmental conditions and demographic composition [8, 9]. Previous studies have reported high levels of resistance in *An*. *funestus* to pyrethroids in Tanzania [10] and elsewhere in East Africa [11–13]. *An*. *funestus* is one of the major malaria vectors in Africa and is widely distributed across the continent [14, 15]. The species is highly anthropophilic and endophilic, with higher vectorial capacity than most other vector species [14, 16]. The *An*. *funestus* group includes several sibling species, which are difficult to distinguish morphologically, but can be separated by polymerase chain reaction (PCR) using a cocktail of species-specific primers [17]. Previous studies in Tanzania have reported presence of four member species in the *An*. *funestus* group, namely *An*. *funestus* s.s, *An*. *rivulorum*, *An*. *leesoni* and *An*. *parensis* [18]. All the four members of the *An*. *funestus* group have been found positive for *P*. *falciparum* though the infection rates are often higher in the predominantly anthropophagic *An*. *funestus* s.s than the rest. The importance of this vector is increasingly associated with the persistent malaria transmission [8, 10], and it has been suggested that it could be responsible for resurgence of malaria transmission in rural Tanzania [10].

In this study, we assessed contributions of residual populations of major malaria vectors, *An*. *arabiensis* and *An*. *funestus*, in endemic villages in rural south-eastern Tanzania, where the last mass distribution of LLINs prior to our study had been completed in 2011 [19]. The study assessed prevalence of *Plasmodium* sporozoites in the vector species, survival rates of these vectors, and their resistance status to examine what factors might be mediating the role of these two major species in the ongoing malaria transmission. Inferences are made with regard to what would happen if appropriate species-specific interventions were deployed in this and similar settings. More specifically, interventions that effectively target mosquito species mediating most of the remnant malaria transmission should result in extensive declines in the transmission. Where one vector species dominates the transmission system, approaches against such vectors may offer opportunities to sufficiently disrupt local malaria transmission.

## Methods

### Study area

**Fig 1:**
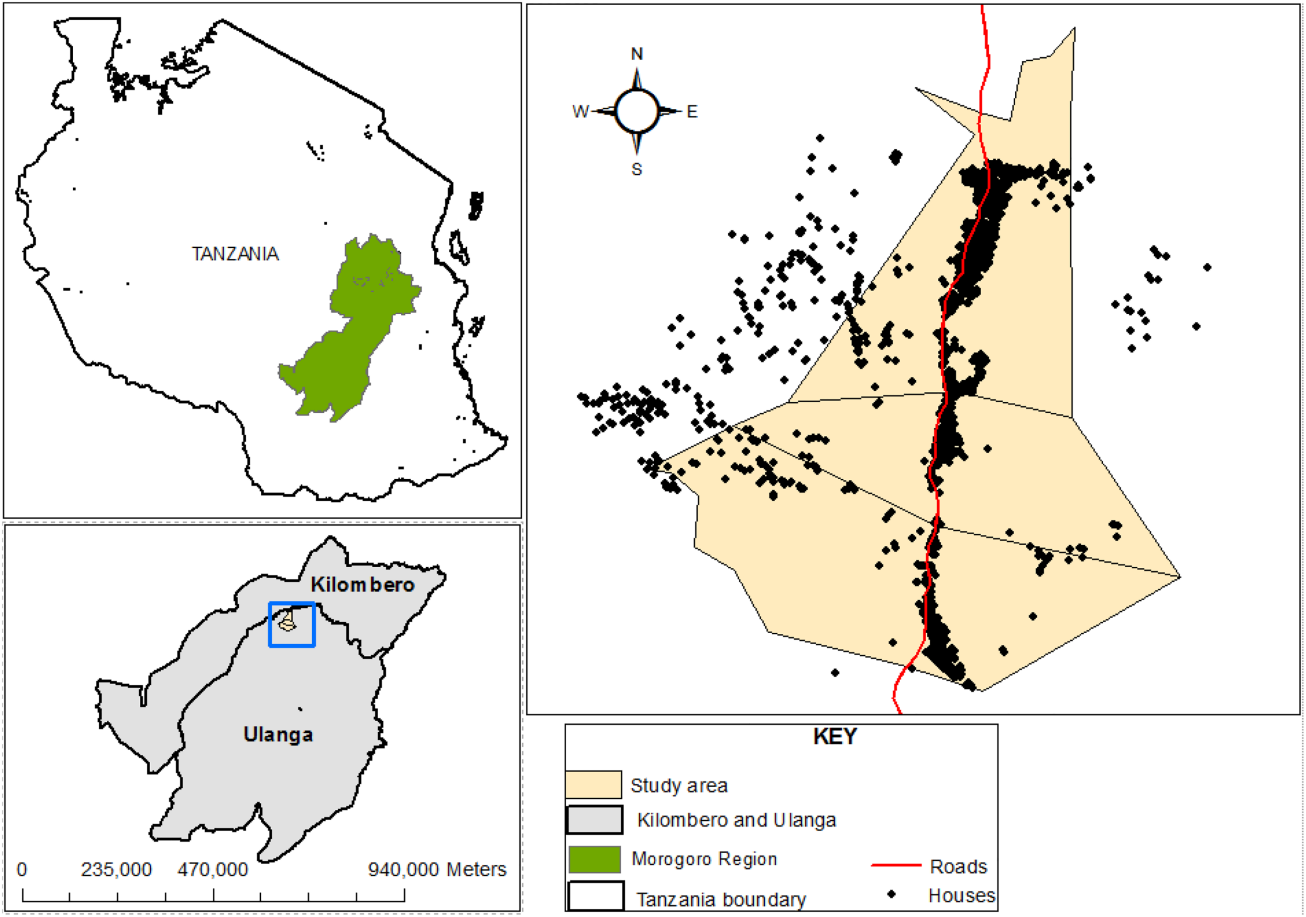
Map of the study area, showing the villages and households in Ulanga district where the study was conducted.

The study was conducted in three villages in the Kilombero floodplains in Ulanga district, south-eastern Tanzania (Fig 1). This area is perennially meso-endemic for malaria and has high mosquito densities throughout the year, peaking between January and May. Annual rainfall and temperatures range from 1200 to 1800 mm and 20°C to 32.6°C respectively. Malaria vector species in the area comprise primarily of the *An*. *gambiae s*.*l* (almost exclusively consisting of *An*. *arabiensis)*, and *An*. *funestus group*. Several other *Anopheles* mosquitoes such as *An*. *coustani*, *An*. *pharoensis*, *An*. *squamosus*, *An*. *ziemanni* and *An*. *wellcomei* are also found, as well as several culicine species, mainly *Mansonia* mosquitoes, *Aedes* mosquitoes and *Culex* species. The major vector control intervention in this area is LLINs [19], and the last mass-distribution of nets, prior to this study had been conducted between 2010 and 2011 [19].

### Study procedures

This study was part of a high-resolution longitudinal mosquito surveillance program, where we collected host-seeking and indoor-resting mosquitoes between January 2015 and January 2016, in randomly selected households, and also selected sentinel households within the Health and Demographic Surveillance System (HDSS) maintained by Ifakara Health Institute in the study area [20].

#### Monitoring indoor densities of host-seeking and resting disease-transmitting mosquitoes

Firstly, 1600 households were randomly selected from the HDSS households database in three study villages of Kivukoni, Minepa and Mavimba, and assigned to 16 geographical clusters each consisting of 100 households. The sampling clusters were assigned based on household latitudes so that clusters 1–16 were arranged in a north-south direction in the study area. From each geographical cluster, six households were selected for sampling. These included three households randomly selected on monthly basis, and another three households that were fixed as sentinel households, visited repeatedly every month. This way, 96 households were studied per month, each time consisting of 48 sentinel households across the study area, and 48 new households randomly selected each month. Geo-positions of all the households were recorded using hand held GPS receivers (Magellan eXplorist 110). Each household was visited only once each month, and mosquitoes were sampled from one room with occupant(s) sleeping under mosquito nets, using miniature Centre for Disease Control (CDC) light traps set near occupied bed nets at the foot end, at approximately 1.5 m from the ground [21, 22]. The traps were operated from18:00 to 06:00 each night.

Indoor resting mosquitoes were collected using a backpack aspirator [23] from the walls, bed nets and floors in same households where the CDC light trap catches were conducted. Aspiration of resting mosquitoes was conducted for approximately 15 minutes in each household. Each morning, the collected mosquitoes were killed in a closed container using alcohol. The mosquitoes were identified morphologically and sorted by taxa and sex before they were identified by PCR. The identification was done following the taxonomic key developed by Gillies and Coetzee, i.e. Keys for the *Anopheles* of Africa, south of Sahara [24].

#### Identification of sibling species and detection of Plasmodium infection in malaria vectors

All female *An*. *gambiae* complex mosquitoes, were examined by multiplex polymerase chain reaction (PCR) technique as described by Scott *et al*. [18] to distinguish sibling species. Similarly, all samples of *An*. *funestus* were examined by PCR, using techniques developed by Koekemoer *et al*. [25] and Cohuet *et al*. [26] to distinguish sibling species. All *Anopheles* mosquitoes caught were segregated by species and examined in pools by enzyme-linked immunosorbent assays (ELISA), to detect *Plasmodium* sporozoites in their salivary glands [27]. To avoid false positives, the ELISA lysates were boiled for 10 minutes at 100°C, effectively eliminating heat-labile non *Plasmodium falciparum* protozoan antigens [28]. We assessed all the collected *Anopheles gambiae* s.l and *Anopheles funestus* s.l mosquitoes by ELISA, using heads and thoraces. However, only a sub-sample was assessed by PCR for species identification, using legs.

#### Identification of mosquito blood meals

Blood meal analysis was also done on a sub-sample, working only with the mosquitoes that were blood-fed. Each mosquito abdomen was separated from the thorax and homogenized in phosphate buffer saline (PBS) following the method described by Beier *et al*. [29]. This way, all the blood-fed mosquitoes were screened for human, bovine, chicken, goat and dog blood using ELISA.

#### Assessment of insecticide susceptibility and parity rates

The insecticide susceptibility tests were conducted using standard WHO guidelines [30]. Insectary-reared *An*. *gambiae* s.s (Ifakara strain) was used as a reference susceptible population. Because of the difficulties in finding *An*. *funestus* larvae in the study area, adult female mosquitoes were collected indoors from April to July 2016, by CDC light traps from the same households in three villages. The mosquitoes were transferred into netting cages and maintained on 10% glucose solution. *An*. *funestus* mosquitoes were then separated from other mosquito species by gently sucking them from the cages into a small-netting cage using mouth aspirators and all other mosquito species were killed.

Non blood-fed adult *An*. *funestus* s.l females were therefore exposed to 0.75% permethrin, 0.05% deltamethrin, 0.05% lambda cyhalothrin, 4% dieldrin, 4% DDT, 0.1% bendiocarb, 0.1% propoxur, 0.25% pirimiphos methyl, and 1% malathion, as recommended by the WHO. Up to 100 adult females of unknown age were exposed to each insecticide during the experiment, including at least four replicates. In each test, mosquitoes collected from the same villages at the same time were exposed to oil impregnated papers, constituting additional controls. The number of knocked-down mosquitoes in each tube was counted and recorded after 10, 15, 20, 30, 40, 50 and 60 minutes. After one hour of exposure, mosquitoes were transferred to the holding tubes and maintained on 10% sugar solution then monitored for survival and mortality after 24 hours. The post exposure observation was conducted after 80 minutes only if the knockdown was less than 80% [30].

Physiological age of the wild-caught female malaria vectors was approximated based on the status of their ovaries, i.e. whether they had previously laid eggs or not [31]. Abdomens of a randomly selected sub-samples of unfed female *An*. *funestus* and *An*. *arabiensis* collected from the selected households were dissected for parity [32]. Each unfed female mosquito was first anesthetized in a refrigerator. A drop of distilled water was added to a slide and each specimen kept still on the slide, then the seventh and eighth abdominal segment was pulled using a fine needle under a stereo dissecting microscope. The ovarial tracheoles were then observed under a compound microscope at 10X magnification, to determine whether they were parous or nulliparous [31].

### Data analysis

Statistical analyses were carried out using R statistical software version 3.3.1 [33]. Mosquito abundance was over-dispersed so we modeled it following a negative binomial distribution using the generalized linear mixed model automatic differentiation model builder (glmmADMB) package [34] with log link function. Here, mosquito abundance (nightly total count) were treated as response variable while locality (villages) were treated as fixed effect.

*Plasmodium* infection rates and blood meal sources of malaria vectors were estimated as percentages of total mosquitoes assayed. Similarly, observed mean mortality after 24 hours of exposure to each insecticide was calculated as percentage of total mosquitoes exposed in all four exposure replicates [30]. The insecticide susceptibility test findings were interpreted according to WHO guidelines [30].

The EIR was calculated as a function of biting rates and proportion of tested mosquitoes infected with sporozoites. Since we initially calculated these biting rates for each trap night, the EIR was adjusted by multiplying the daily EIR values by 365 nights (Annual EIR = Nightly biting rate x Sporozoite rate x 365). We relied on values comparative trap evaluations done in the same study area, where CDC light traps had been directly compared to human landing catches inside households [35]. Those earlier trials had indicated that CDC light traps consistently caught 0.3 times as many *An*. *gambiae* s.l as human landing catches, and 0.68 times as many *An*. *funestus* mosquitoes as human landing catches [35]. In this current study, we have therefore adjusted the CDC light trap catches using these coefficients, but also presented the unadjusted results.

### Ethical considerations

Meetings with local leaders were held in the study areas and the main aims of the study were explained by the scientists. Permission to conduct studies in the separate villages was obtained from respective leaders of each of the villages; Mr. Ayubu Salila, Mr. Kiluka Mdumba and Mr. Baalesa Ligola for Kivukoni, Minepa and Mavimba villages respectively. I confirm that the field studies did not involve endangered or protected species. A written and signed informed consent was obtained from each household head participating in the study. All information was given in Kiswahili, the local language. All the households were provided and protected by intact LLINs (Olyset^®^nets) as previously distributed by the universal bednets coverage campaign [36]. Ethical approval for the study was obtained from Ifakara Health Institute Institutional Review Board (IHI/IRB/No: 34–2014), and the Medical Research Coordination Committee of the National Institute of Medical Research (Certificate No.NIMR/HQ/R.8a/Vol.IX1903).

## Results

### Indoor densities of *Anopheles* mosquitoes in the area

**Fig 2:**
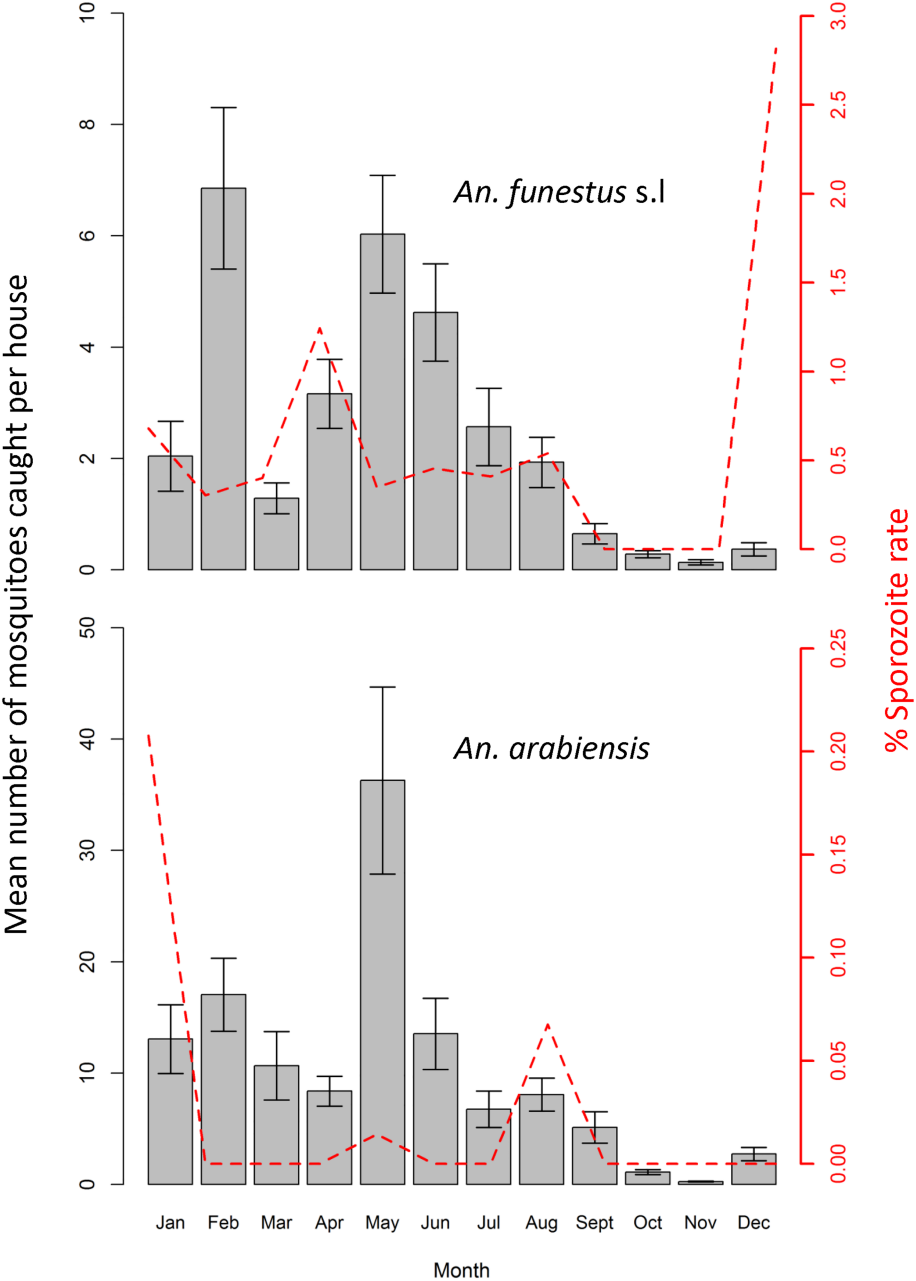
Mean number of *Anopheles funestus* s.l *and Anopheles arabiensis* mosquitoes caught per house per night. Y error bars represent the 2-standard errors of the mean.

A total of 25670*Anopheles* mosquitoes were collected by CDC light trap and back pack aspirator, comprising *An*. *funestus* s.l (18.7%) and *An*. *gambiae* s.l, which in turn comprised of *An*. *arabiensis* sibling species (79.0%), *An*. *pharoensis* (0.5%), *An*. *squamosus* (0.4%), *An*. *ziemanni* (0.3%) and *An*. *wellcomei* (0.4%). The highest densities, i.e. 54% and 65% of all *An*. *arabiensis* and 65% of all *An*. *funestus* were observed in the middle of study area, i.e. Minepa village. Densities of *An*. peaked between May, while the density of *An*. *funestus* peaked in February and May.one month after onset of the long rainy season, which is the main malaria transmission season. The lowest vector densities were observed between September and November (Fig 2).

### Sibling species of malaria vectors and *Plasmodium* infection rates

**Fig 3:**
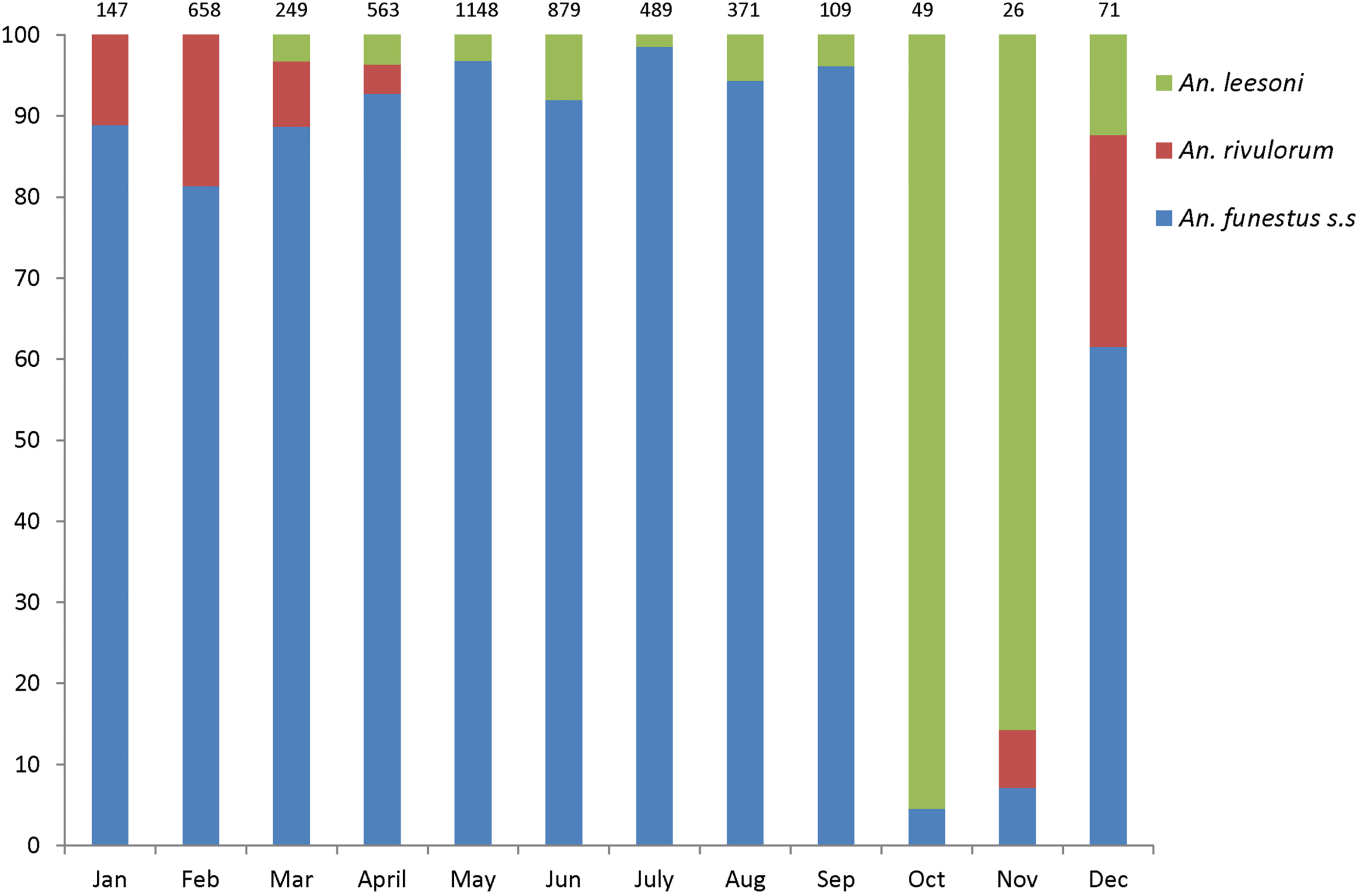
Monthly variations in the composition of sibling species of *Anopheles funestus* group in the study area.

A total of 20,135 *An*. *gambiae* s.l and 4,759 *An*. *funestus* s.l mosquitoes were assayed in the laboratory by PCR or ELISA or both. All the *An*. *gambiae* s.l. mosquitoes tested by PCR were confirmed to be *An*. *arabiensis* (100%). On the other hand, the *An*. *funestus* group consisted of 76.6% *An*. *funestus* s.s, 2.9% *An*. *rivulorum*, 7.1% *An*. *leesoni*, and 13.4% unamplified samples. Monthly variations of proportions of the *An*. *funestus* sibling species is shown in Fig 3.

The CDC-light trap catches were used to estimate malaria transmission densities as Entomological Inoculation Rates (EIR) as shown in Table 1. Infection rates were higher *in An*. *funestus* than *An*. *arabiensis*. Using unadjusted estimates, the overall EIR (bites per person per year) was 9.19, of which *An*. *funestus* contributed 86.21%, while *An*. *arabiensis* contributed 13.79% (Table 1). However, when considering estimates adjusted on the basis of relative efficiencies of CDC light traps compared to Human Landing Catches, the overall EIR (bites per person per year) was 15.87, of which *An*. *funestus* contributed 73.39%, while *An*. *arabiensis* contributed 26.61% (Table 1). The highest sporozoite rate among *An*. *funestus* was in April, when 13.79% towards the transmission. Nightly biting rate among *An*. *funestus* was highest in May, i.e. 344 bites/month, while the highest biting rate in *An*. *arabiensis* was 2090 bites/month in the same month (Table 2). Of all *An*. *funestus* s.l mosquitoes that tested positive for malaria parasites, 96% were *An*. *funestus* s.s, the remaining 4% being *An*. *rivulorum*. None of the *An*. *leesoni* tested positive for *Plasmodium* sporozoites.

**Table 1:** Infectious status of malaria vectors and dominant contribution of *Anopheles funestus* mosquitoes to ongoing malaria transmission, despite their low abundance relative to *Anopheles arabiensis*.

	*Anopheles arabiensis*	*Anopheles funestus s*.*l*
Total number of mosquitoes collected by CDC Light Trap (January 2015 to January 2016)	20135	4759
Total number of trap nights	1152	1152
Biting rate per night	17.48	4.13
Relative efficiency (CDC-LT) relative to HLC (Derived from Okumu et al 2008 [38])	0.3	0.68
Corrected biting rate	58.26	6.08
Total number of mosquitoes analysed for *Plasmodium falciparum* circumsporozoite protein (CSP)	20135	4759
Total number of sporozoite positive mosquitoes	4	25
Sporozoite rate	0.0002	0.0053
Annual EIR (Adjusted)**	**4.22**	**11.65**
% EIR Contribution (Adjusted)**	**26.61%**	**73.39%**
**Annual EIR (not adjusted)**	**1.27**	**7.92**
**% EIR Contribution (not adjusted)**	**13.79%**	**86.21%**

• Annual EIR (Corrected biting rate x Sporozoite rate x 365)

• Overall EIR (EIR for *An*. *arabiensis*+ EIR for *An*. *funestus)* = 18.45

• *86.3% of the mosquitoes from the *An*. *funestus group* tested were *An*. *funestus s*.*s*, 8.4% were *An*. *leesoni* and 5.2% were *An*. *rivulorum*. Of all *An*. *funestus* s.l mosquitoes that tested positive for malaria parasites, 96% were *An*. *funestus s*.*s*, the remaining 4% being *An*. *rivulorum*.

• **Where the estimates are adjusted, these adjustments were done using coefficients computed as the ratio of mosquito catches by CDC-Light Traps to catches Human Landing Catch methods. These coefficients were 0.3 for *An*. *arabiensis* and 0.68 for *An*. *funestus* as determined by Okumu et al 2008 [35]

**Table 2:** Monthly sporozoite rates and Entomological Inoculation Rates (EIR) (number of infectious bites per person per month) for *Anopheles funestus* and *Anopheles arabiensis* from January to December 2016.

*Anopheles funestus*					*Anopheles arabiensis*			
Month	Number of Mosquitoes Tested	Monthly biting rates (adjusted**)	Sporozoite Infection Rate	Mean Monthly EIR	Number of Mosquitoes Tested	Monthly biting rates (adjusted **)	Sporozoite Infection Rate	Mean Monthly EIR
January	147	44.10	0.0219	0.97	963	288.90	0.0068	1.96
February	658	197.40	0.0107	2.12	1640	492.00	0.0000	0.00
March	249	74.70	0.0129	0.96	2111	633.30	0.0000	0.00
April	563	168.90	0.0413	6.98	1502	450.60	0.0000	0.00
May	1148	344.40	0.0113	3.89	6968	2090.40	0.0005	0.94
June	879	263.70	0.0153	4.04	2576	772.80	0.0000	0.00
July	489	146.70	0.0132	1.94	1283	384.90	0.0000	0.00
August	371	111.30	0.0174	1.94	1478	443.40	0.0023	1.00
September	109	32.70	0.0000	0.00	859	257.70	0.0000	0.00
October	49	14.70	0.0000	0.00	244	73.20	0.0000	0.00
November	26	7.80	0.0000	0.00	51	15.30	0.0000	0.00
December	71	21.30	0.0910	1.94	460	138.00	0.0000	0.00

** Values adjusted based on Okumu et al 2008; see Table 1

### Indoor densities of Culicine mosquitoes in the area

A total of 103,382 Culicine mosquitoes, comprising of 100,047 *Culex* (96.7%), 3197 *Mansonia* (3.1%) and 138 *Aedes* mosquitoes (0.1%). Of all the *Culex* mosquitoes collected, males contributed 76.3%, while females contributed 23.7%. For *Mansonia*, males contributed 58.6% while females contributed 41.4%. Lastly, *Aedes* males contributed 26.1% while females were 73.9%.

### Mosquito blood meal sources

A total of 82 mosquitoes that were identified by PCR also had blood meals in their abdomen, so these were assessed by way of blood-meal ELISA. These included 64 *An*. *arabiensis*, 17 *An*. *funestus* s.s, and 1 *An*. *leesoni*. The study identified a broad spectrum of host blood antigens, mostly humans (79.2%), followed by cattle (17.1%), dogs (2.4%) and chicken (1.2%). The analysis did not detect any mixed blood meals. The results for mosquito blood meals are summarized in Table 3.

**Table 3:** Host blood antigens detected in blood-meals obtained from *Anopheles* mosquitoes.

Blood meal source	*Anopheles arabiensis*		*Anopheles funestus* s.s		*Anopheles leesoni*	
	N	%	N	%	N	%
Human	47	73.4	17	100	1	100
Bovine	14	22	0	0	0	0
Dog	2	3.1	0	0	0	0
Chicken	1	1.5	0	0	0	0

### Spatial distribution of sporozoite infected *An*. *funestus* and *An*. *Arabiensis*

**Fig 4:**
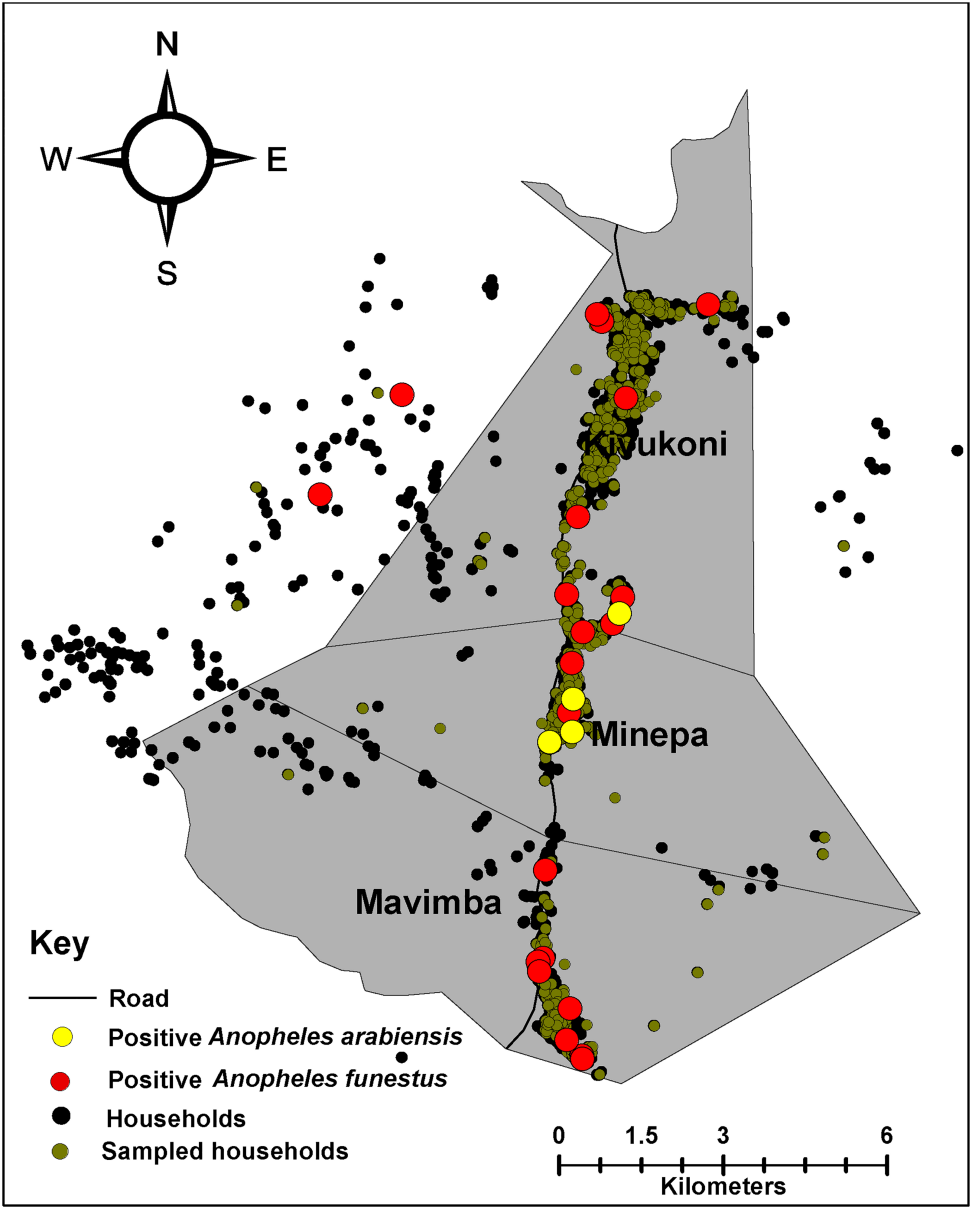
Distribution of *Plasmodium* infected *An*. *funestus* and *An*. *arabiensis*.

Sporozoite infected *An*. *funestus* were widely distributed across all the study villages, it was also observed that *An*. *arabiensis* were mostly confined to an area in the middle of the study area (Fig 4). The total number of sporozoite infected mosquitoes during the year was, however, too small to enable detailed analysis of spatial relations.

### Insecticide susceptibility test results

The insecticide susceptibility test results are summarized in Table 3. There was 100% mortality in the susceptible reference *An*. *gambiae s*.*s* colony when exposed to all the candidate insecticides tested. The wild-caught *An*. *funestus* were also fully susceptible to pirimiphos methyl (100% mortality), malathion (100%), and dieldrin (100%), but they were resistant to permethrin (10.5% mortality), deltamethrin (18.7%), lambda cyhalothrin (18.7%) and DDT (26.2%). In these tests, the *An*. *funestus* also showed reduced susceptibility to bendiocarb (95% mortality) and propoxur (90.1% mortality). The overall mortality of the *An*. *funestus* mosquitoes exposed to control papers was 5.4%.

### Parity rates of *An*. *funestus* relative to *An*. *arabiensis* mosquitoes

Of the 302 *An*. *funestus* dissected, 65.8% were parous, 28.8% were nulliparous, and 5.2% were pre-gravid. Of the 162 *An*. *arabiensis* dissected, 49.3% were parous, 37% were nulliparous and 13.5% were pre-gravid.

## Discussion

Malaria prevalence is declining across most of African countries [1], due to improved vector control [1] and case management, but also urbanization, improved health care and better living standards [1]. Long-lasting insecticide treated nets (LLINs) and indoor residual spraying (IRS) contributed 78% of all gains accrued since 2000 [37]. In rural south-eastern Tanzania, where malaria prevalence has reduced by >60% since 2000, low-level transmission still persists despite persistently high LLIN coverage [38]. Our study re-assessed the residual malaria transmission dynamics in rural south-eastern Tanzania, in an area where the last mass distribution of bednets was conducted between 2010 and 2011, four years before our study [19]. This study therefore provides an essential update, of the transmission situation and demonstrates the now dominant role of *An*. *funestus*. Like in most residual transmission settings in East Africa, our study confirms that populations of formerly notorious *An*. *gambiae* s.s, have significantly diminished, or completely vanished [10, 39]. *An*. *arabiensis* now dominates in numbers, but the far more competent vector *An*. *funestus* carries most of the residual malaria parasites, despite being highly outnumbered by *An*. *arabiensis*. This study clearly provides a clear lead to the hypothesis that interventions targeting *An*. *funestus* could dramatically reduce malaria transmission in areas such as the ones where this study was conducted.

This study has also determined that *An*. *funestus* vector is resistant to pyrethroids used on LLINs (Table 4), and survives unexpectedly longer than *An*. *arabiensis*, thus requiring new control approaches. Given the extensive resistance in the species population against a wide range of insecticides currently approved for public health use, it appears that we now have only a limited set of options, including organophosphates such as pirimiphos methyl. Thus, one of the main control methods that could be used against this species is focal IRS with long-lasting formulations of organophosphates (e.g. Actellic^®^ CS-300), against which *An*. *funestus* are still highly susceptible. Other potential interventions may include house improvement and house screening, as well as use of eave baffles treated with organophosphates, which would take far less insecticide, but deliver equal impact on the mosquito populations as IRS (Killeen *et al* unpublished manuscript). Going forward, it may be desirable to conduct new evidence-based studies in well-characterized areas such as these study sites, to demonstrate possible vector elimination strategies leading to malaria transmission control. Approaches may include combinations of organophosphate-based IRS alongside other interventions like LLINs, improved housing and larval source management, especially where rice growing is commonly practiced.

**Table 4:** Summary of data on susceptibility of wild-caught adult female *Anopheles funestus* mosquitoes collected in the study sites.

Insecticide tested	Total. No. Exposed	Number of mosquitoes knocked- down (KD)								Mortality		
		10 min	15 min	20 min	30 min	40 min	50 min	60 min	% KD 60 min	Total No. Dead	% Dead	Status*
Control	129	0	0	0	0	0	0	0	0	7	5.4	**-**
0.75% Permethrin	95	0	0	0	0	1	1	1	1.1	10	10.5	RR
0.05% Deltamethrin	80	2	2	1	2	3	3	6	7.5	15	18.7	RR
0.05% Lambda cyhalothrin	80	0	0	1	1	1	2	4	5.0	15	18.7	RR
0.1% Bendiocarb	80	0	2	2	6	27	31	35	43.7	76	95.0	RI
0.1% Propoxur	81	21	36	46	67	58	75	77	95.0	73	90.1	RI
0.25% Pirimiphos-methyl	84	6	3	8	46	63	80	80	95.2	84	100.0	SS
5% Malathion	80	7	13	32	55	74	78	79	98.7	80	100.0	SS
4% DDT	80	0	0	4	7	9	8	13	16.2	21	26.2	RR
0.4% Dieldrin	80	0	0	0	0	2	6	14	17.5	80	100.0	SS

*Susceptibility status:

• SS: Indicates susceptibility

• RR: Confirmation of resistance in the tested mosquito population.

• RI: Suggest the existence of resistance and further investigation is needed.

To achieve malaria elimination, we must identify and target all pockets of transmission, including geographically distinct areas of high transmission and demographically high-risk sub-populations [40, 41]. In this study setting in rural south-eastern Tanzania, low-level transmission persists, despite over 80% LLIN coverage [38]. The persistent residual transmission is most definitely mediated predominantly by *An*. *funestus* s.s, suggesting possibly that targeted, high impact, species-specific interventions may improve control and accelerate efforts towards eventual malaria elimination. Our recent findings suggested that the overall distribution of malaria mosquitoes, including *An*. *funestus* is strongly influenced by and can be predicted based on numbers and spatial variability of household occupancy [8]. The densities of *Anopheles* mosquitoes were highest in rainy season even though transmission was evidently sustained at low densities even in dry season, mostly by *An*. *funestus*. Since aquatic breeding habitats for *An*. *funestus* are not easily identified, it may be necessary to identify other environmental characteristics that affect natural abundance of *An*. *funestus* within villages and between households. This approach can be feasible by involving the local community and volunteers. The evidence from Mwangungulu *et al* show that scientists can rely on community knowledge and experiences to identify areas where mosquitoes are most abundant [42].

The data presented here would be essential in developing control measures that target primarily the most important residual vector species, in this case *An*. *funestus*. In these specific study settings in Tanzania, the nucleated settlements, and spatial correlations between human biomass and vector densities [8], indicates this proposed study area renders itself well to spatially-targeted interventions. The study has rigorously demonstrated that *An*. *funestus* is now the primary vector of malaria in this area in rural-south eastern Tanzania, despite concurrently high densities of other species such as *An*. *arabiensis*. The area historically had very high malaria transmission, averaged 400 infectious bites/person/year (ib/p/y) until early 2000s [43], with prevalence as high as 70% in 1990s [44], but this study indicates that transmission has now dropped around 20 ib/p/y. Moreover, this study has also demonstrated that *An*. *funestus* s.s co-exists with *An*. *rivulorum* and *An*. *leesoni*, but malaria transmission was mainly mediated by *An*. *funestus* s.s. It is not possible to determine conclusively if the greater contribution of the *An*. *funestus* s.s. to malaria transmission is mostly due to its relatively higher abundance than that of the other sibling species, but it is clear that this species mediates most of the residual transmission.

Of all the *An*. *funestus* mosquitoes that tested positive for malaria parasites, *An*. *funestus* s.s. comprised 96% of those *An*. *funestus* s.l. which were positive for *Plasmodium* sporozoites., though their overall relative abundance was 76.6%, while *An*. *rivulorum* was responsible for 4% of the infections though their relative abundance was only 2.9% of all the *An*. *funestus* mosquitoes. *Anopheles leesoni*, constituted 7.1% of all mosquitoes in this group, but carried no infections at all. Given that the ELISA lysates were all boiled to eliminate any false positives [28], occasionally associated with non-*Plasmodium* protozoans transmitted by zoophilic mosquitoes [45], we can assume, based on the 4% sporozoites rates, that *An*. *rivulorum* is indeed an important vector in this setting, and that its role is limited mostly by its low densities, relative to the other species. Other studies in East Africa have previously demonstrated the importance of the species in residual transmission settings [46], its role increasing after the decline of *An*. *gambiae* s.s populations [47, 48] (Table 5). Our study did not however, identify any *Plasmodium* infections in any of the other *Anopheles* mosquitoes collected, i.e. *An*. *coustani*, *An*. *squamosus*, *An*. *ziemanni* and *An*.*wellcomei*, at any time during the study, thus these species are likely to be of negligible importance in malaria transmission in this setting. Furthermore, this can be attributed to their low relative abundance in this area. Some previous studies have reported sporozoite infections in *An*. *coustani* [49], *An*. *squamosus* [50] and *An*. *ziemanni* [51], but no such evidence was observed in this current study. Table 5 shows some of the other localities where *An*. *funestus* mosquitoes significantly contribute to the residual malaria transmission.

**Table 5:** Examples of other localities where *Anopheles funestus* mosquitoes have been demonstrated to mediate most of the residual malaria transmission, and where control programs targeting *An*. *funestus* with effective interventions, could drastically reduce local transmission.

SN	Country	Intervention	Dominant Vectors	Other Vectors	Resistance Status	Sporozoite Rates	Implications for malaria transmission control	Reference
1	Kenya	ITNs and LLINs	*An*. *funestus*	*An*. *arabiensis*, *An*. *gambiae* s.s.	Pyrethroid resistance confirmed	*An*. *funestus* 4.5%, *An*. *arabiensis* 0.9%, *An*.*gambiae* s.s 8.6%	Reduced effectiveness of the current interventions	[52]
2	Burkina Faso		*An*. *gambiae* s.l.	*An*. *funestus* s.l, *An*. *nili*	No pyrethroid detected. *An*. *funestus* in was highly resistant to dieldrin	*An*. *funestus* (2.6% -9.7%)	*An*. *funestus* had higher biting rates and high sporozoite rate compared to *An*.*gambiae* complex	[53]
3	Tanzania	LLINs	*An*. *gambiae* s.l.	*An*. *funestus* s.l	Pyrethroid resistance confirmed	*An*. *funestus* (0.16% -1.47%)	Resurgence of malaria transmission in Kilombero valley, Tanzania	[10]
4	Senegal	LLINs	*An*. *funestus*	-	-	*An*. *funestus* 1.28%	*An*. *funestus* changed their host seeking behaviour following the introduction of LLINs, thus compromising effectiveness of LLINS.	[54]
5	Cameroon		*An*. *funestus* s.s	*An*. *gambiae* s.s	-	*An*. *funestus* 6.8%, *An*.*gambiae* s.s 0.6%- 4.1%	*An*. *funestus* accounts for 88% of the transmission in this setting.	[55]

The extremely high proportion of *Plasmodium* infections currently mediated by *An*. *funestus* mosquitoes in this area is compounded by, and is likely also a result of the very high insecticide resistance in this species. This study found that *An*. *funestus* mosquitoes were not only carrying most of the residual malaria parasites, but they were also very highly resistant to common pyrethroids used for vector control, i.e. deltamethrin, permethrin, lambda-cyhalothrin, as well as to the organochlorine, DDT. Moreover, there was also reduced susceptibility to insecticides like bendiocarb and propoxur, which would normally be candidates for replacing the pyrethroids. An earlier study by Lwetoijera *et a*.*l* in the neighbouring Kilombero district, also found increasing importance of *An*. *funestus* in malaria transmission associated with high resistance [10]. Even though the insecticide susceptibility status for *An*. *arabiensis* was not evaluated in this study, a separate study from these villages by Matowo *et al* (unpublished findings) found *An*. *arabiensis* to be also highly resistant to pyrethroid chemicals.

While the high human blood index observed in the *An*. *funestus* clearly suggests that the species could still be best targeted by household-based interventions that protect humans. Observations of increasing outdoor biting by the different *An*. *funestus* sibling species has been reported in this study area [56, 57], which warrants consideration of complementary interventions that also target outdoor-biting vectors. Also, given the high resistance to the common insecticides for vector control, it appears that use of organophosphates, against which the vector is still susceptible, notably pirimiphos methyl, could provide a temporary solution against residual malaria transmission in this area. Long-lasting formulations of pirimiphos methyl already exist [58], which could be used for IRS in this area to complement LLINs, as it has already been implemented in Zanzibar, northern region and lake zones in Tanzania [59].

Other possible high value interventions could be insecticidal eave baffles (Killeen et al. Unpublished), especially in areas where even outdoor-biting populations forage indoors for their adult life [60]. This study did not assess the role of *An*. *funestus* on outdoor malaria transmission, but, a separate study conducted in the same villages by Ngowo *et al* (unpublished findings) yield evidence that some *An*. *funestus* collected outdoors were found to be sporozoite positive. Furthermore, tools should be included such as chemicals with new AIs available for IRS that are being evaluated for consideration by the WHO, including non-pyrethroid active ingredients like neonicotinoids as well as bednets that combine synergists with pyrethroids, or those that combine different insecticide classes to tackle insecticide resistance and lower vector survival.

One limitation of this study is that collections for insecticide susceptibility were conducted on wild caught females of variable age, since we could not find adequate larvae for the assays. WHO recommends where possible to use mosquitoes of the same age cohort (F1 progeny). Using wild mosquitoes that are older than the standard 3–5 days may lead to underestimation of resistance depending on the species distribution and the insecticide being tested. Blood meal analyses were carried out on visually blooded mosquitoes and so may have missed those with small amounts of blood in their abdomens, such as those that had interrupted feeding or had partially digested the blood. Another potential limitation is that parity assessments, such as the ones conduced here are greatly impacted by season, this study conducted parity assessment for mosquitoes collected for two months, we suggest that future further studies should be done to assess parity status across seasons. Lastly, there was also a significant non-amplification of mosquito samples during the PCR analysis to determine the sibling species. About 13.4% of all sample analyzed in the laboratory did not amplify. An analysis of the sample handling processes, the analysis process, and the control samples, suggests that the laboratory processes were unlikely to be the reason for the non-amplification. Instead, this situation might have arisen due to sub-optimal storage conditions of some samples, or minor discrepancies in the morphological identification. These specific processes therefore deserve greater attention in future analyses.

## Conclusion

This study provides an update on the residual malaria transmission situation in rural south-eastern Tanzania, where LLINs were already widely distributed. Though *An*. *arabiensis* is still the most abundant vector species here, ongoing residual transmission is predominantly mediated by *An*. *funestus*, possibly due to high resistance and high survival probabilities. Interventions that effectively target *An*. *funestus* could therefore significantly improve control of residual malaria transmission in the area.

## Supporting information

S1 File Supplementary data. A file containing supplementary data depicting graphs used in this manuscript.
(CSV)
